# Supplementary material for: Prediction of Long-Term Stroke Recurrence Using Machine Learning Models
Source: J Clin Med. 2021 Mar 20;10(6):1286. doi: 10.3390/jcm10061286 (PMC8003970; doi:10.3390/jcm10061286)
Supplement: Supplementary file 1 [file jcm-10-01286-s001.pdf]

Table S1. Feature selection applied to cases and controls based on four criteria. Set 1: all features; Set 2: all features except medication history; Set 3: features selected by at least two data-driven strategies; Set 4: minimum set, obtained as the intersect of Set 2 and Set 3.

		Control vs. Case group 1		Control vs. Case group 2		Control vs. Case group 3		Control vs. Case group 4		Control vs. Case group 5	
**Complete Feature set (SET 1)**	**SET 2**	**SET 3**	**SET 4**	**SET 3**	**SET 4**	**SET 3**	**SET 4**	**SET 3**	**SET 4**	**SET 3**	**SET 4**
Age	X	X	X	X	X	X	X	X	X	X	X
Sex	X	X	X	X	X	X	X	X	X	X	X
Body mass index	X	X	X	X	X	X	X	X	X	X	X
Diastolic blood pressure	X	X	X	X	X	X	X				
Systolic blood pressure	X	X	X	X	X	X	X	X	X	X	X
Hemoglobin	X	X	X	X	X	X	X	X	X	X	X
Hemoglobin A1c	X			X	X	X	X	X	X	X	X
High-density lipoprotein	X	X	X	X	X	X	X	X	X	X	X
Low-density lipoprotein	X	X	X			X	X	X	X		
Platelet	X	X	X	X	X	X	X	X	X		
White blood cell	X	X	X	X	X	X	X	X	X		
Creatinine	X	X	X	X	X	X	X	X	X	X	X
Smoking	X	X	X	X	X	X	X	X	X	X	X
Difference in days between the index date and the last outpatient visit prior to the index stroke				X		X		X			
MEDICAL HISTORY											
Atrial fibrillation or flutter	X					X	X	X	X	X	X
Atrial fibrillation						X		X		X	
Atrial flutter						X		X		X	
Chronic heart failure	X	X	X	X	X	X	X	X	X	X	X
Chronic kidney disease	X	X	X	X	X	X	X	X	X	X	X
Chronic liver disease	X					X	X				
Chronic liver disease (mild)						X		X		X	
Chronic liver disease (moderate to severe)											
Chronic lung disease	X	X	X			X	X				
Diabetes	X	X	X	X	X	X	X	X	X	X	X
Dyslipidemia	X			X	X	X	X	X	X	X	X
Hypertension	X	X	X	X	X	X	X	X	X	X	X
Myocardial infarction	X	X	X			X	X				
Neoplasm	X										
Hypercoagulable	X										
Peripheral vascular disease	X	X	X	X	X	X	X	X	X	X	X
Patent foramen ovale	X			X	X	X	X				
Rheumatic disease	X					X	X				
MEDICATION HISTORY											
Aspirin						X		X			
Antihypertensives		X		X		X					
Clopidogrel						X		X			
Coumadin/Warfarin						X					
Dipyridamole		X		X		X		X		X	
Other oral anticoagulants		X		X		X		X		X	
Statins		X		X		X		X		X	
MEDICATION at DISCHARGE											
ACE inhibitors						X					
Angiotensin receptor blockers						X					
Apixaban/Rivaroxaban		X				X		X			
Aspirin											
Beta blockers						X					
Calcium channel blockers				X		X					
Clopidogrel											
Coumadin/Warfarin		X		X		X		X		X	
Dabigatran											
Dipyridamole											
Diuretics				X		X					
Statins		X		X		X		X		X	
FAMILY HISTORY											
Heart disorder	X			X	X	X	X				
Stroke	X										

Table S2. Comprehensive model performance measures for the 288 prediction models. Link: https://github.com/TheDecodeLab/GNSIS_v1.0/tree/master/ModelingStrokeRecurrence/SupplementaryMaterials.

Table S3. Feature importance ranking for the different modeling frameworks.

Features Abbreviations	Gradient Boost Mean (SD)	Random Forest Mean (SD)	XGBoost Mean (SD)	Decision Trees Mean (SD)	SVM Mean (SD)	Logistic Regression Mean (SD)	Overall Average Mean (SD)
Age	96.91(7.22)	99.88(0.45)	95.35(11.15)	98.38(5.68)	94.34(12.49)	57.82(14.23)	90.45(4.83)
Body mass index (BMI)	71.95(11.58)	79.89(8.11)	35.13(18.05)	65.33(36.76)	42.51(11.46)	50.99(12.56)	57.63(9.82)
creatinine	43.41(20.94)	58.22(10.74)	28.98(29.83)	64.04(39.08)	61.31(13.93)	27.68(9.17)	47.27(11.29)
Current Smoker	2.44(1.63)	5.39(3.09)	0.7(1.5)	32.96(39.64)	7.15(4.69)	49.39(7.8)	16.34(13.69)
Diastolic Blood Pressure	40.65(10.69)	59.98(6.26)	12.54(13.64)	49.2(37.98)	11.44(8.49)	8.81(8.53)	30.44(10.89)
High-density lipoprotein (HDL)	55.87(11.46)	69.25(5.69)	30.3(16.44)	64.8(34.84)	31.9(7.53)	41.85(16.26)	49(9.76)
Hemoglobin A1C	63.86(15.58)	72.34(10.86)	27.84(19.91)	59(35.07)	46.32(8.13)	21.33(10.04)	48.45(9.47)
Hemoglobin	56.5(10.87)	70.09(5.72)	25.66(16.53)	49.13(39.92)	48.12(9.75)	13(9.63)	43.75(11.47)
Last outpatient visit	70.7(23.85)	73.06(7.97)	28.8(27.68)	75.03(36.43)	3.38(3.63)	18.98(12.16)	44.99(11.81)
Low-density lipoprotein (LDL)	65.51(14.7)	73.21(6.12)	25.73(16.66)	45.75(39.17)	11.83(7.27)	15.48(18.42)	39.59(11.06)
Male	1.99(1.34)	8.1(3.42)	0.6(1.26)	2.07(6.22)	18.82(9.76)	0(0)	6.31(3.61)
Platelet	71.6(14.45)	74.59(5.9)	19.18(20.5)	50.19(38.05)	6.63(7.29)	12.22(7.83)	39.07(11.28)
Systolic Blood Pressure	58.2(12.29)	72.03(7.76)	28.37(20.33)	62.01(36.31)	25.73(8.6)	28.89(12.26)	45.87(10.06)
White blood cell	71.09(15.8)	76.21(6.53)	29.14(22.74)	54.36(37.42)	10.11(11.3)	5.81(7.69)	41.12(10.83)
Atrial fibrillation or flutter	1.79(1.93)	4.08(1.26)	0.08(0.29)	71.98(40.56)	5.31(3.3)	22.15(8.64)	17.57(14.42)
Atrial fibrillation	0.39(0.32)	7.42(2.19)	1.15(1.34)	25.15(33.9)	21.48(9.38)	6.68(6.45)	10.38(11.69)
Atrial flutter	3.4(1.57)	8.09(2.77)	2.19(2.47)	42.23(39.9)	22.85(10.53)	10.65(7.65)	14.9(13.47)
Chronic liver disease (mild)	1.23(2.13)	3.99(1.7)	0.27(0.75)	30.17(42.85)	6.69(3.54)	7.21(15.08)	8.26(15.18)
Chronic liver disease (mod/severe)	0(0)	0.64(0.54)	0(0)	0.04(0.1)	0.99(0.53)	1.21(1.62)	0.48(0.57)
Diabetes	9.34(3.69)	13.64(4.57)	20.51(11.01)	68.25(35.25)	54.17(20.75)	34.49(16.01)	33.4(10.85)
Dyslipidemia	4.12(1.87)	10.52(2.85)	1.3(1.98)	29.33(36.22)	34(15.05)	7.55(9.5)	14.47(12.17)
Heart failure (CHF)	5.38(2.96)	7.59(4.04)	3.23(4.14)	52.44(39.94)	25.88(11.51)	18.82(13.8)	18.89(12.84)
Hypercoagulable	0.13(0.34)	2.51(0.52)	0.01(0.02)	5.68(12.63)	1.21(0.94)	7.97(7.15)	2.92(4.77)
Hypertension	3.15(2.67)	8.67(3.43)	1.3(2.57)	39.88(40.23)	32.06(12.29)	15.04(8.79)	16.69(13.31)
Kidney diseases	6.91(3.85)	11.54(5.28)	13.45(13.11)	61.84(41.87)	41.3(20.11)	28.64(18.68)	27.28(12.69)
Liver diseases	3.36(4.07)	3.48(2.22)	0.41(1.33)	47.06(43.76)	6.17(3.73)	17.06(18.08)	12.92(15.29)
Lung diseases	4.77(2.33)	10.24(2.89)	2.69(3.21)	51.53(36.69)	18.68(4.71)	15.14(7.99)	17.17(12.32)
Myocardial infarction	3(1.52)	7.42(2.37)	0.75(1.16)	42(40.51)	16.23(7.54)	7.51(5.66)	12.82(14.08)
Neoplasm	4.68(4.25)	10.4(2.67)	1.94(3.84)	48.43(43.36)	12.76(2.7)	17.49(11.18)	15.95(14.72)
Patent Foramen Ovale	5.06(2.05)	9.14(3.14)	1.8(2.87)	54.61(35.13)	12.65(4.95)	40.22(21.43)	20.58(12.5)
Peripheral vascular disease	4.84(2.45)	9.35(3.46)	4.38(5.9)	50.06(38.98)	30.7(10.73)	23.7(10.03)	20.5(12.5)
Rheumatic diseases	1.47(1.1)	4.19(2.39)	0.51(1.79)	38.18(42.87)	7.57(3.33)	17.08(8.97)	11.5(15.08)
Anti-hypertensives	9.25(6.59)	12.93(5.5)	7.8(8.88)	68.81(38.26)	15.69(5.22)	42.47(13.55)	26.16(11.64)
Aspirin	3.19(1.42)	6.42(1.91)	0.38(0.57)	55(41.59)	13.49(5.78)	6.76(5.06)	14.21(14.73)
Clopidogrel	0(0)	6.38(2.07)	0.66(1.15)	1.24(4.12)	13.49(5.78)	0(0)	4.36(2.32)
Coumadin/Warfarin	2.64(1.97)	6.54(1.74)	0.36(0.58)	45.07(37.73)	13.22(5.46)	6.13(3.55)	12.33(13.35)
Dipyridamole	0(0)	2.27(1.14)	0(0)	1.55(2.74)	3.97(1.88)	0.17(0.1)	1.33(1.05)
Oral anticoagulants	0(0)	0.82(1.37)	0(0)	0.26(0.75)	2.52(1.15)	0.09(0.09)	0.62(0.56)
Statins	5.85(1.8)	11.76(2.33)	3.64(3.52)	44.56(36.35)	34.52(12.93)	16.09(11.04)	19.4(12)

ACE Inhibitors	2.11(1.17)	6.53(1.16)	0.78(1.45)	66.7(37.74)	11.61(5.84)	15.11(7.44)	17.14(13.17)
Angiotensin Receptor Blockers	0(0)	0.25(0.24)	0(0)	0.23(0.73)	2.41(1.01)	0.7(1.1)	0.6(0.45)
Apixaban/Rivaroxaban	0(0)	1.35(1.13)	0(0)	2.35(3.69)	3.13(1.21)	5.68(4.77)	2.08(1.81)
Aspirin discharge	5.1(4.01)	11.36(1.58)	2.4(4.3)	52.97(42.67)	11.7(10.23)	11.8(8.25)	15.89(14.14)
Beta Blockers	2.3(2.03)	6.51(1.6)	0.12(0.28)	63.93(37.98)	7.59(5.04)	14.67(12.57)	15.85(13.29)
Calcium Channel Blockers	2.16(1.34)	3.68(1.04)	0.09(0.29)	72.34(37.64)	3.39(2.3)	28.77(5.86)	18.4(13.57)
Clopidogrel Discharge	2.55(1.96)	7.86(1.87)	0.53(1.39)	58.34(39.61)	5.29(3.26)	20.24(7.71)	15.8(13.89)
Coumadin/Warfarin Discharge	8.46(5.81)	9.83(6.23)	13.45(10.33)	84.35(31.45)	31.02(4.27)	60.6(10.58)	34.62(9.26)
Dabigatran	0(0)	0.61(0.38)	0(0)	0.14(0.37)	0.69(0.5)	2.56(2.48)	0.67(0.86)
Dipyridamole Discharge	0.31(0.65)	3.31(0.81)	0.16(0.43)	39.67(49.08)	2.85(1.63)	5.81(4.06)	8.68(18.17)
Diuretics	1.59(2.61)	4.58(1.46)	0.18(0.41)	38.3(48.72)	5.76(2.94)	29.98(12.62)	13.4(17.36)
Statins Discharge	5.49(2.31)	9.33(2.22)	3.09(4.38)	53.76(40.1)	27.49(11.34)	23.66(9.34)	20.47(13.23)
Heart disorder	6.88(4.39)	14.15(3.62)	3.69(5.91)	49.55(42.39)	11.91(12.71)	19.63(8.95)	17.63(13.51)
Stroke	3.33(2.28)	10.77(2.27)	0.76(1.43)	37.07(39.22)	10.09(6.44)	7.07(5.15)	11.51(13.57)
